# *Thismia belumensis* (Thismiaceae), a remarkable new species from The Royal Belum State Park, Gerik, Perak, Peninsular Malaysia

**DOI:** 10.3897/phytokeys.172.59336

**Published:** 2021-02-18

**Authors:** Mat Yunoh Siti-Munirah, Zainol Suhaimi-Miloko, Mohammad Ismail Zubir Ahmad

**Affiliations:** 1 Forest Research Institute Malaysia, 52109, Kepong, Selangor, Malaysia Forest Research Institute Malaysia Kepong Malaysia; 2 No 49, Taman Desa Damai, 33300, Gerik, Perak, Malaysia Taman Desa Damai Gerik Malaysia; 3 The Royal Belum State Park, Perbadanan Taman Negeri Perak, Hentian Amanjaya Royal Belum, Pulau Banding, 33200, Gerik, Perak, Malaysia The Royal Belum State Park Gerik Malaysia

**Keywords:** Conservation status, endemic, Peninsular Malaysia, Perak, *Thismia belumensis*, zygomorphic

## Abstract

This report describes *Thismia belumensis* Siti-Munirah & Suhaimi-Miloko, a novel species of achlorophyllous herb discovered in the Royal Belum State Park, Peninsular Malaysia. This new species is unlike any previously described species of *Thismia*. In particular, *T. belumensis* possesses a unique annulus, which has been expanded and modified into a cucullate (hood-like) structure. This structure covers the apical floral tube and has an opening on one side facing a thickened part of the annulus, and the off-centre floral aperture confers a zygomorphic symmetry to the flower, indicating *T. belumensis* is more similar to *Thismia labiata* J.J.Sm. This morphological detail makes this new species distinct from all other described species of *Thismia*. In this report, we provide descriptions, illustrations, colour plates, and the provisional conservation status of *Thismia belumensis*.

## Introduction

*Thismia* Griff. (Thismiaceae) is a genus of small mycoheterotrophic herbs, currently comprising approximately 80–90 species (Dančák et al. 2020a; Nuraliev et al. 2020; Shepeleva et al. 2020). Plants of this genus are primarily distributed in the tropical regions of Asia, Australia, and South America, and extend into the subtropical and temperate regions of Japan, New Zealand, Australia, and the USA (Merckx et al. 2013; Dančák et al. 2020b). In Peninsular Malaysia, little is known about most *Thismia* species as they are normally only noticeable when in flower, and the flowers can be small, fragile, and ephemeral. The plant is also easily dehydrated and can rapidly degrade if taken from its original habitat. Studying these plants is challenging, as careful and rapid care must be taken to retain the plant’s true appearance and structure. Due to their fast degradation, *Thismia* species cannot be handled and studied like typical pressed herbarium specimens. Therefore, to obtain accurate images of their morphology, they must be dissected in the field, or immediately stored in an airtight container for transport to the research station for further investigation, or immediately preserved in 70% ethanol.

For many decades, the knowledge of *Thismia* species in Peninsular Malaysia has not been updated, with Jonker (1948) the most recent taxonomic. Seventy years later, in 2018, a new species description was published – *Thismia kelantanensis* Siti-Munirah (Siti-Munirah 2018). Since then, two more species have been described – *Thismia domei* Siti-Munirah and *Thismia terengganuensis* Siti-Munirah (Siti-Munirah and Dome 2019). It is likely that more novel species will be discovered in the future (Siti-Munirah and Dome 2019). The most recent publication describing Malaysian *Thismia* is from Sarawak (Borneo) and describes *Thismia minutissima* Dančák, Hroneš & Sochor (Dančák et al. 2020a). Overall, throughout Malaysia, there are currently approximately 30 *Thismia* species, of which – including *T. belumensis*, described in this report, – 14 of these are found in Peninsular Malaysia. These are: *Thismia alba* Holttum ex Jonker, *Thismia arachnites* Ridl., *Thismia aseroe* Becc., *Thismia chrysops* Ridl., *Thismia clavigera* F. Muell., *Thismia crocea* (Becc.) J.J.Sm, *Thismia domei* Siti-Munirah & Dome, *Thismia fumida* Ridl., *Thismia grandiflora* Ridl., *Thismia javanica* J.J. Sm, *Thismia kelantanensis* Siti-Munirah, *Thismia racemosa* Ridl. and *Thismia terengganuensis* Siti-Munirah (Jonker 1948; Siti-Munirah 2018; Siti-Munirah and Dome 2019).

**Map 1.**
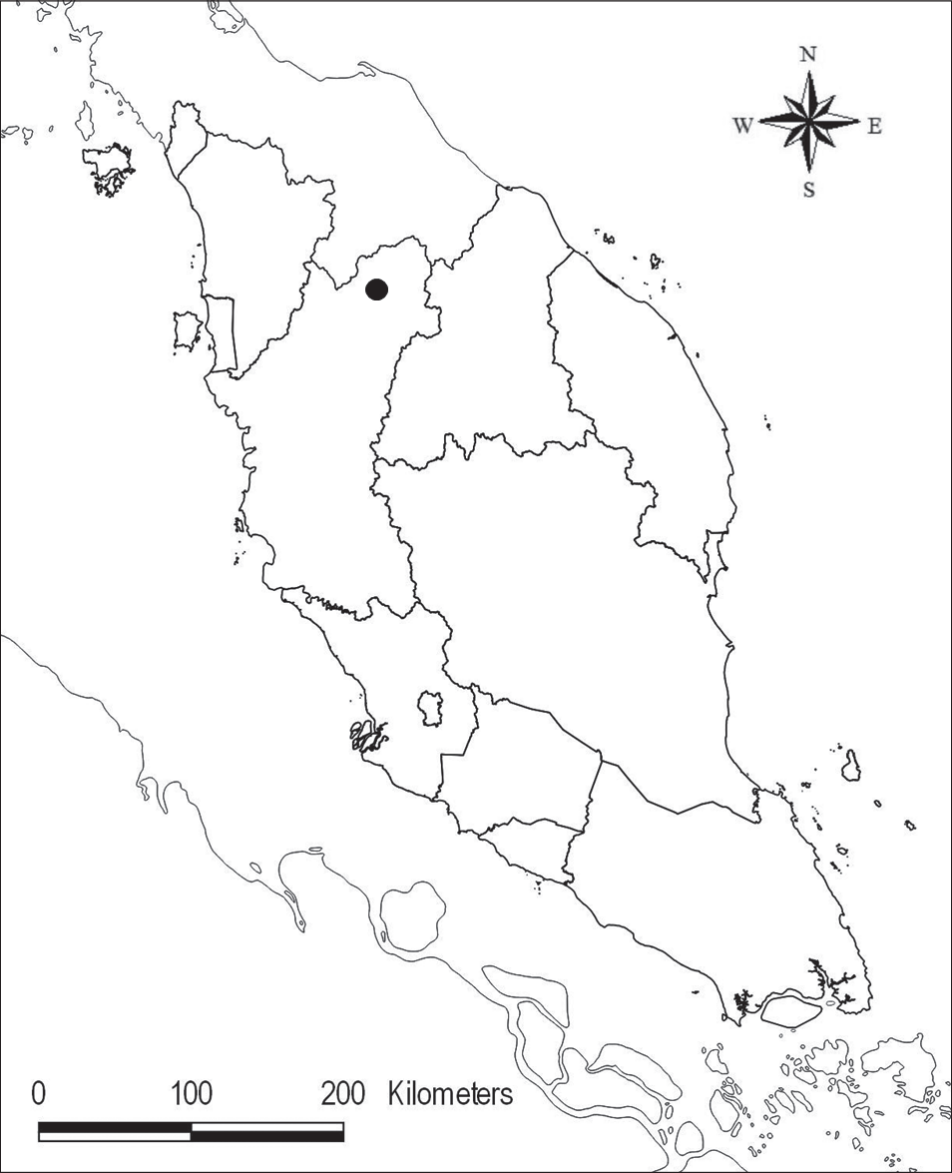
Map of Peninsular Malaysia indicating the location of *Thismia belumensis* (●).

The *Thismia* species described here was first discovered in 2017 by Mr. Suhaimi-Miloko during a guided nature tour in the Royal Belum State Park (SP), Perak, Peninsular Malaysia (Map 1). The majority of the Royal Belum SP is covered by pristine primary tropical rainforest and was established as a 117,500 ha forest park in 2007 by the Perak state government. Royal Belum SP is the second-largest protected area in Peninsular Malaysia – after the Taman Negara (431,435 ha) (Schwabe et al. 2015). The new species of *Thismia* was discovered in the Sungai Gadong forest area, in the southern part of the Royal Belum SP. This location is also an important habitat of *Rafflesia azlanii* Latiff & Wong, and *R. cantleyi* Solms, which possess the world’s largest flowers (Siti-Munirah 2012, 2020). In October 2019, we visited the site, and several specimens of the new *Thismia* species were collected for taxonomic study. After careful examination, some novel characteristics of the annulus and tepals were identified in the specimens. These traits formed a unique combination of characteristics that did not match any described species of *Thismia*. Hence, it is described here as a new species.

## Materials and methods

This study is based on material collected by M.Y. Siti-Munirah in October 2019 from The Royal Belum SP, Gerik. The specimens were preserved in 70% ethanol in the Kepong (KEP) herbarium collections. Morphological characteristics were studied using a stereomicroscope and high-resolution macro photography. Measurements were taken from both living and alcohol-preserved material. The specimen details were thoroughly compared with drawings and descriptions in the protologues of *Thismia* species worldwide.

## Taxonomic account

### Thismia belumensis

Taxon classificationPlantaeDioscorealesBurmanniaceae

Siti-Munirah & Suhaimi-Miloko sp. nov.

BEB14980-415C-569A-ABF5-8E7BD04DA4D9

urn:lsid:ipni.org:names:77215193-1

Figs 1 , 2 , 3

#### Diagnosis.

Most similar to *Thismia labiata* J.J.Sm. but differing in the floral tube partially covered by a massively expanded cucullate bilabiate annulus, longer outer tepals appendage, obovate to spathulate rather than linear to filiform inner tepals and the supraconnective apex curved outwards like a skirt rather than straight.

**Figure 1.**
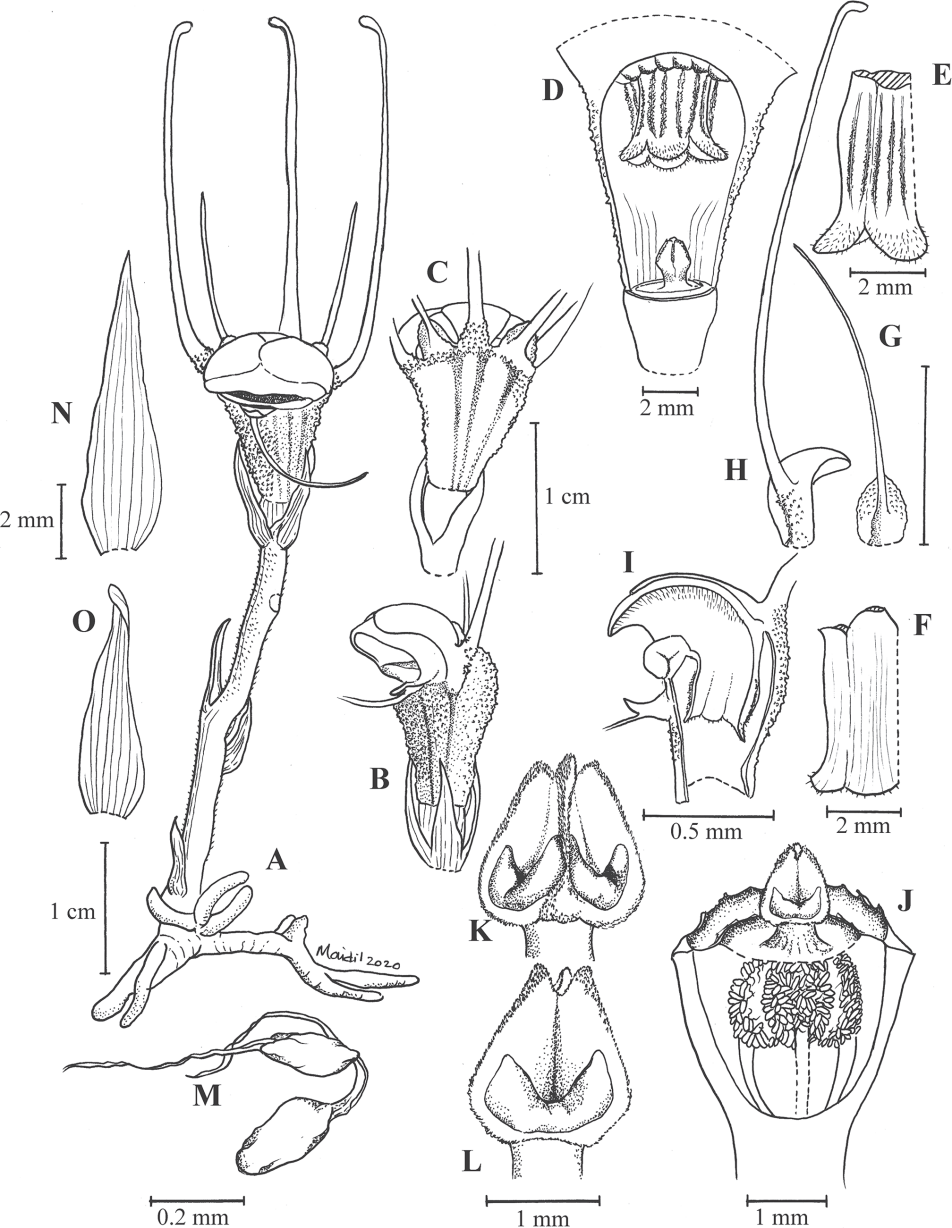
Illustration of *Thismia belumensis* Siti-Munirah & Suhaimi-Miloko **A** habit **B** side view of flower **C** back view of flower **D** longitudinal section of the floral tube showing pendulous stamen with ovary and stigma **E** outer view of stamens **F** inner view of stamens **G** outer tepal **H** inner tepal **I** longitudinal section of hood (annulus) and pendulous stamens **J** longitudinal section of ovary **K** stigma **L** pistil **M** ovules **N** bract **O** leaf. All from *FRI 94752* & *FRI 94758*, drawn by Mohamad Aidil Noordin.

#### Type.

Malaysia. Peninsular Malaysia: Perak, Gerik Distr., Royal Belum State Park, ca. 290 m alt., 22 October 2019, *M.Y. Siti-Munirah FRI 94758* (holotype: KEP!, spirit collection, No. barcode 279998).

**Figure 2.**
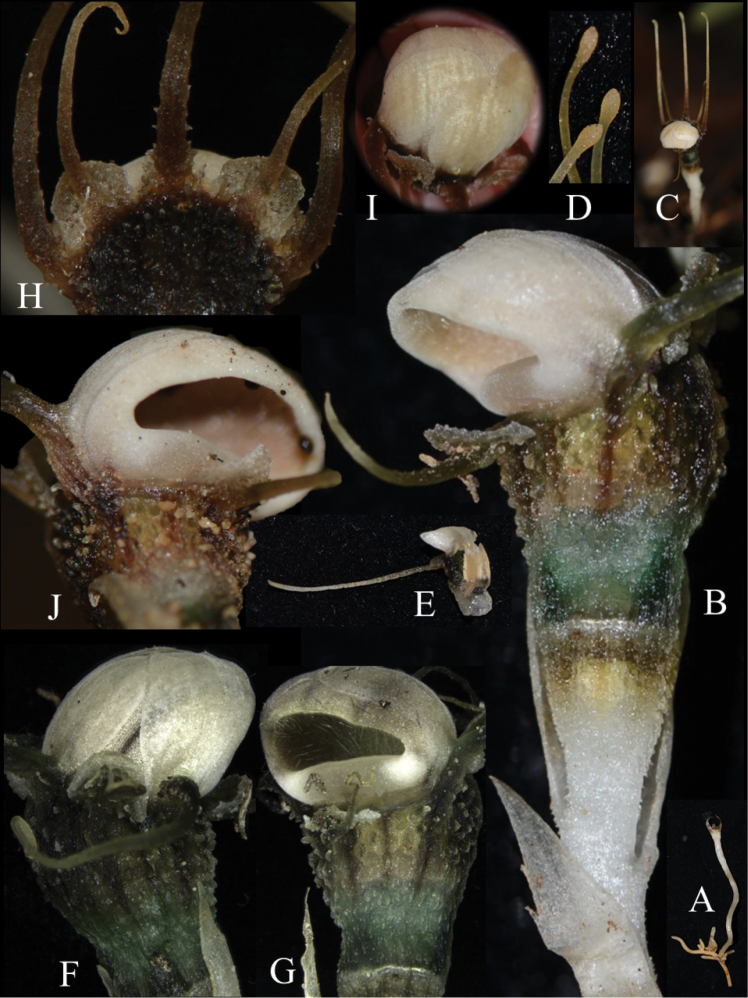
Outer appearance of *Thismia belumensis* Siti-Munirah & Suhaimi-Miloko **A** root and stem **B** flower from side view **C** flower showing the erect tepal appendages of the live plant **D** tip of erect tepal appendages **E** slender appendage of outer tepal **F** side view showing inner tepals overlaying the cucullate structure **G** floral tube with the floral aperture of the expanded annulus **H** outer tepals alternate with inner tepals, each with distal or dorsal appendage **I** top view of flower showing inner tepals overlapping on cucullate structure **J** semi-round shape of the aperture of the annulus (All photos by Siti-Munirah MY, *FRI 94752* & *FRI 94758*).

#### Description.

Terrestrial, achlorophyllous, brownish-whitish-green herbs up to 8 cm tall. ***Roots*** vermiform white-brownish. ***Stems*** erect (sometimes ascending), unbranched, 2–4 cm long, glabrous. ***Leaves*** scale-like, simple, translucent white, 5 mm long, 1–2 mm wide, triangular, apex acute or acuminate, base appressed. ***Involucral bracts*** 3, white, up to ca. 1 cm long, lanceolate, apex acute to acuminate, margin entire, glabrous, base appressed. ***Pedicel*** 2–4 mm long (post anthesis). ***Flowers*** terminal, zygomorphic, solitary, 5–8 cm long (including appendages); ***floral tube* (hypanthium)** 5–7 mm long, 3–6 mm wide, narrowed just above the ovary ca. 3 mm wide, widest on upper part ca. 6 mm wide, slightly shorter at one side (lower on the floral aperture side), ***outer surface*** verrucose covered with very short minute warts (papillae); basal half green, apical half with 12 dark brown and 12 pale brown (almost translucent) vertical stripes; ***inner surface*** smooth without transverse bars and other ornamentation; emerald green and translucent; ***outer tepals*** 3, pale brown each 3 × 2 mm, apex acute, each with forming a distal filiform, tentacle-like appendage, the two on the opposite side of the annulus opening erect and the one below the thickened annulus slender, ca. 1.5–2.5 cm long, ca. 1 mm wide, cylindrical, brownish-greenish and sometimes whitish towards the subulate tip; ***inner tepals*** 3, brownish to pure white, glabrous (smooth), obovate to spathulate, tightly adpressed and almost completely overlapping the cucullate part of annulus, basally with long and erect appendages, ca. 3 cm long, brownish-greenish and sometimes whitish towards clavate tip. ***Annulus*** expanded and modified into a cucullate (hood-like) structure and thickened part of the annulus, the cucullate covering the apical part of the floral tube and forming a downwards floral aperture and facing thickened part of the annulus on one side of the flower; cucullate outer surface white with 3 black lines, glabrous; cucullate inner surface white to brownish-peach, covered with numerous white translucent trichomes pointing inwards. ***Stamens*** 6, pendent from the apical part of the floral tube; each connective ca. 3.3 mm long, peach; outer surface with two linear to filiform thecae, each 1.8 mm long, facing the inner wall of the floral tube; inner surface smooth; supraconnective apex blunt and rounded, curved (like a skirt) outwards, covered with transparent trichomes on margin (apparent only in the living state); lateral appendage, apical appendages and interstaminal glands absent; ***filaments*** short, connected to floral tube and annulus. ***Ovary*** inferior, unilocular, cup-shaped, ca. 3 mm × 4.7 mm, whitish brown outer surface covered with numerous warts; ***placentas*** 3, bearing numerous ovules; ***style*** ca. 0.4 mm long; ***stigma*** triangular-pyramidal, dark greenish, ca. 1.55 mm long, papillate, unusual whitish thickened part on each surface (sometimes difficult to see), apex truncate (trilobed). ***Fruit*** cup-shaped, white or pale brown with white operculum, pedicel not elongated.

**Figure 3.**
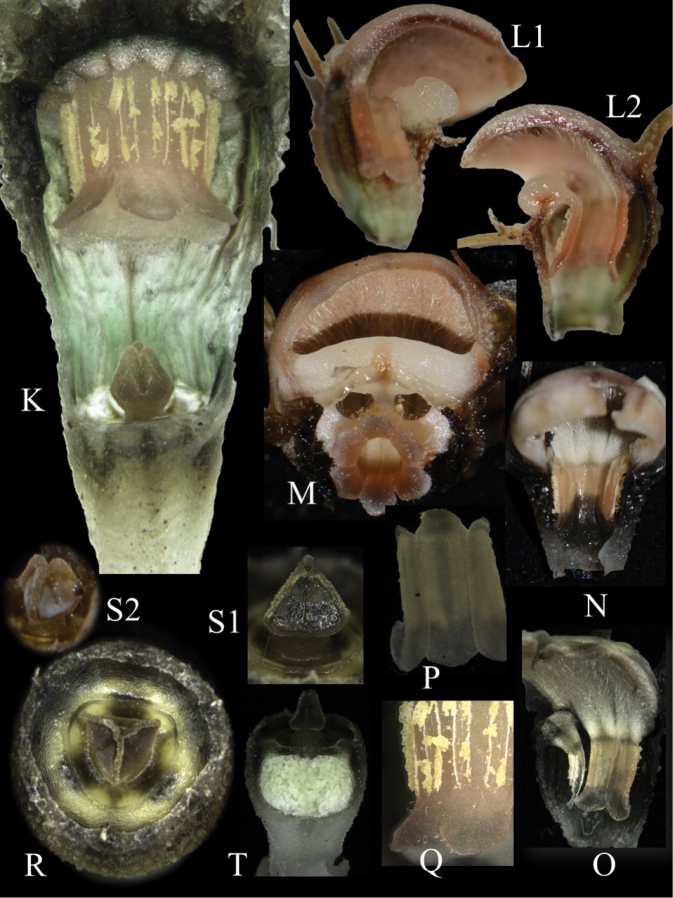
Inner flower appearance of *Thismia belumensis* Siti-Munirah & Suhaimi-Miloko **K** longitudinal section showing the internal parts of flower **L** longitudinal section of floral tube and apical part (**L1** left side **L2** right side) showing pendulous stamen attached to the filament, expanded annulus developing a cucullate (hood-like) structure, thickened part of the annulus at one-side, and outer surface of hood overlaid by inner tepals **M** transverse section of floral tube at lower part showing the apex of fused pendulous stamen and the floral aperture with the thickened part of the annulus in the centre **N** inner view of stamens and filament attached to the expanded annulus, which is covered with white trichome hairs **O** inner view of two stamens and hood inner surface **P** stamen (from inner view) **Q** stamen displaying linear to filiform thecae (outer view) and supraconnective apex curved outwards **R** top view of ovary and stigma **S** stigma from side view (**S1** shows triangular-pyramidal **S2** shows whitish part on surface) **T** cross-section of ovary showing young seeds (All photos by Siti-Munirah MY, *FRI 94752* & *FRI 94758*).

#### Distribution.

Endemic to Perak, Peninsular Malaysia. Currently known only from the type locality (Map 1).

#### Ecology.

**Figure 4.**
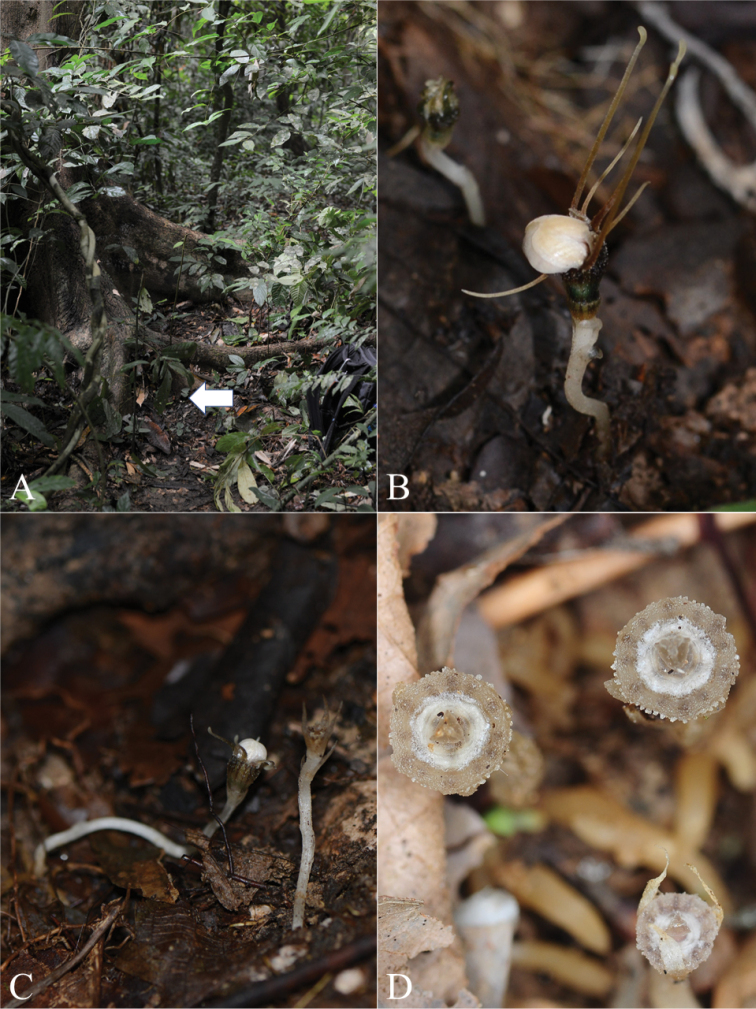
*Thismia belumensis* Siti-Munirah & Suhaimi-Miloko **A** habitat **B***T. belumensis* growing in leaf litter, *FRI 94758***C** habit of the young flowering plant and the young fruit of *T. belumensis***D** fruits of *T. belumensis*, in-situ (Photos by **A–C** Siti-Munirah MY **D** Suhaimi-Miloko Z).

**Figure 5.**
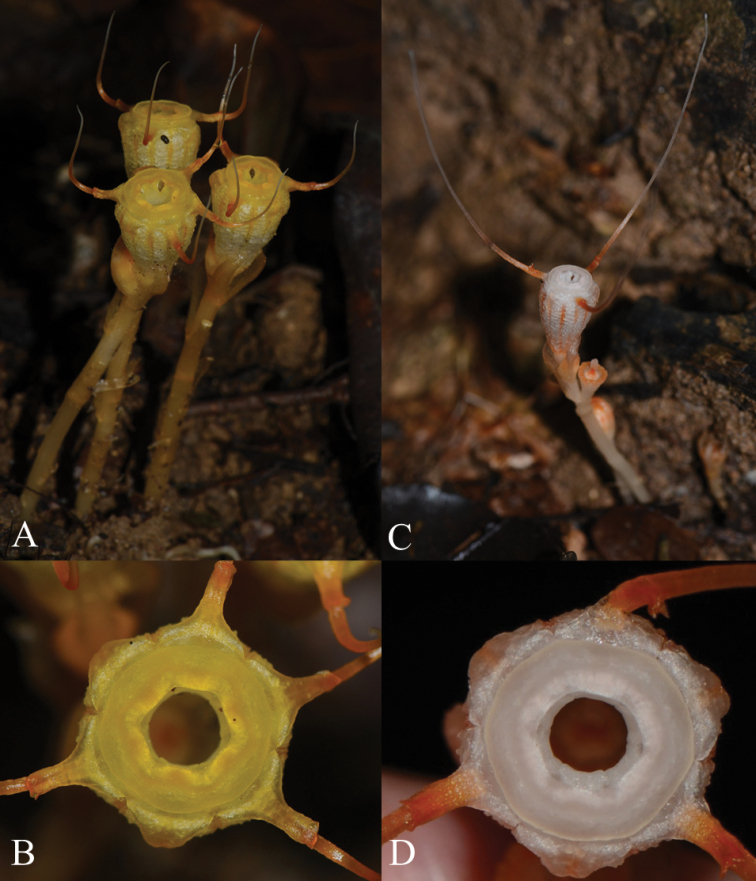
Other *Thismia* species found in the same area as *T. belumensis***A***Thismia* sp. 1, undescribed species **B** top view of *Thismia* sp1. showing the annulus and tepals **C***Thismia javanica***D** top view of *T. javanica* showing the annulus and tepals (symmetrical flower) (All photos by Siti-Munirah MY).

Lowland dipterocarp forest, under shade, 260–290 m altitude. Flowering from June to October. *Thismia belumensis* was found growing within tree leaf litter and between the buttress roots of large trees (Fig. 4). Two other *Thismia* species were also discovered within a radius of about 350 meters, *T. javanica* and *Thismia* sp. 1 (see Fig. 5), which is currently suspected by the authors to be *Thismia arachnites* Ridl., but further examination is still needed. All plants were found close to the walking trail.

#### Etymology.

The species is named after The Royal Belum State Park, the type locality of this species.

#### Conservation status.

Critically Endangered (B1B2ab(iii)). Following the IUCN Standards and Petitions Committee (2019), this species is assessed as critically endangered as it is only known from the type locality and is certainly endemic and rare. Fewer than ten specimens were observed, including flowering and fruiting individuals. Although the locality is within the State Park forest reserve, a protected area, the area is located beside the walking trail, which is a common visiting site for tourists in the Park. The site where *T. belumensis* was found is currently designated as a Tourist Zone in the Royal Belum State Park Management Plan 2018–2027. All guides can freely bring tourists to the area without special permission from the Perak State Park Corporation. Due to the small size of *T. belumensis*, it may not be noticed, and so has a high chance of being stepped on. Without official reminders or guidance, the existence of *T. belumensis* might be threatened by ecotourism activities. *Thismia belumensis* qualifies for CR (B1B2ab(iii)) for its single location, EOO less than 100 km^2^, and AOO less than 10 km^2^. Its habitat quality is also threatened by wild boars (personal observation by Suhaimi-Miloko) and other destructive activities, such as regular visits by tourists to the area. Together, these have the potential to cause a population reduction.

#### Additional specimen examined.

Peninsular Malaysia. Perak: Gerik, Royal Belum State Park, ca. 260 m alt., 22 October 2019, *M.Y. Siti-Munirah FRI94752* (KEP, spirit collection, No. barcode 279997).

## Discussion

This report represents the first description of a novel, notable, and very rare plant – *T. belumensis* – from The Royal Belum SP, Perak, Malaysia. This new species is significantly different from other “fairy lantern” species found in the Malay Peninsula and Borneo. Morphologically, *T. belumensis* is strikingly different from other species by its annulus structure, tepals with an appendage, and floral colours. Nuraliev et al. (2020), describe the annulus as a large, fleshy ring-like structure. However, *T. belumensis* possesses an unusual form of annulus. *T. belumensis* is characterised by most of the annulus being hugely expanded and modified into a cucullate (hood-like) structure covering the apical part of the floral tube, with an aperture on one side facing a thickened part of annulus. The off-centre floral aperture changes the flower symmetry to bilateral, or zygomorphic. In this way, *T. belumensis* is unlike all other known *Thismia* species in Malaysia, which have radially-symmetrical flowers in which the annulus, when developed, has an apical aperture (Fig. 5).

**Figure 6.**
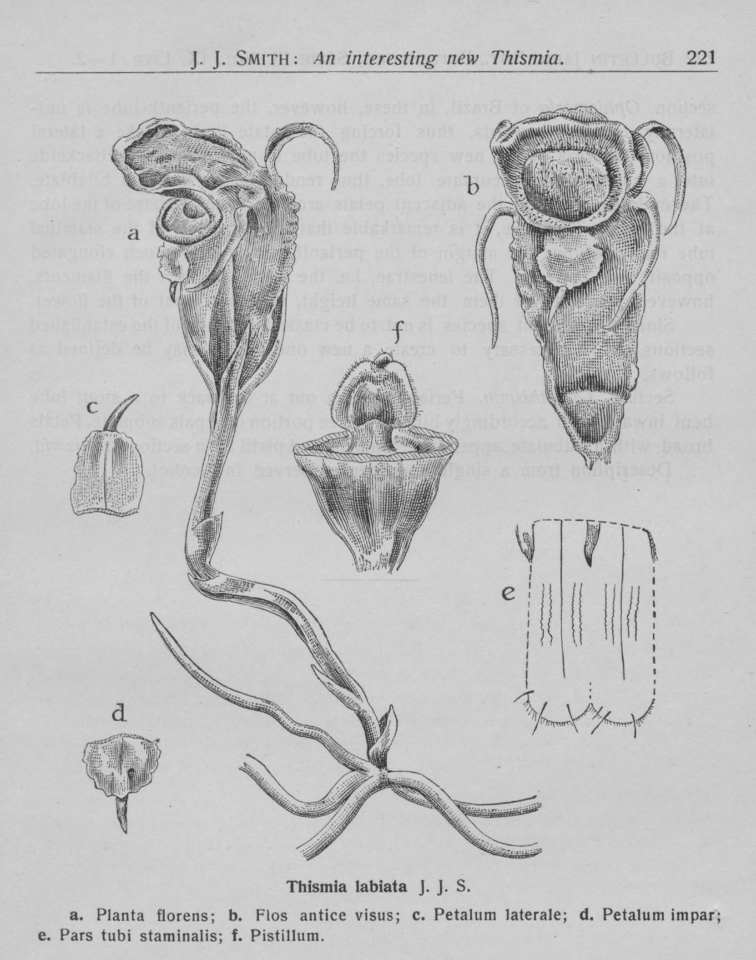
Drawing from the original protologue of *Thismia labiata* (Smith 1927: 221) **A** plant blossoming **B** flower in front view **C** lateral tepal (outer tepal) **D** odd tepal (outer tepal) **E** part of the stamen tube **F** pistil.

*Thismia belumensis* is morphologically similar to *Thismia labiata* J.J.Sm. (Smith 1927) and *Thismia sahyadrica* Sujanapal, Robi & Dantas (Sujanapal et al. 2017). All three species share several unique characters, such as a zygomorphic flower with a sideways facing annulus orifice (*T. belumensis* and *T. labiata*), filiform and linear thecae (*T. belumensis*, *T. labiata* and *T. sahyadrica*), a curved supraconnective (*T. belumensis* and *T. sahyadrica*), and a lack of a lateral appendage and interstaminal glands (*T. belumensis*, *T. labiata* and *T. sahyadrica*). However, most other morphological characteristics of *T. belumensis* are unique. Both *T. belumensis* and *T. labiata* have two types of tepals and appendages; however, these differ between the species. *Thismia belumensis* tepals are unique for the false mitre formed by the inner tepal (loose mitre, if the cucullate structure is absent) (Fig. 2F). With the presence of the cucullate structure, the inner tepals of *T. belumensis*, therefore, completely overlay the outer surface of the cucullate structure (Fig. 2F, I). In *T. labiata*, the annulus forms a labiate structure – a thick, fleshy, upper lip bent over the opening of the floral tube (Fig. 6A), with one inner and two outer tepals of the back of this lip (Fig. 6A, B). There are two more inner tepals between the two lips – one on either side of the flower – and the third outer tepal is inserted in the middle of the lower lip (Fig. 6A, B). In contrast, the outer tepal of *T. belumensis* appears upright (Figs 1G, 2H) with the inner tepals (with erect appendages, Fig. 2C), and with only one slender tepal and appendage (Fig. 2E), attached below the thickened part of the annulus (Figs 2B, 3L2). Both types of tepal in *T. belumensis* have their own form of appendage, and both were much longer compared to appendages of *T. labiata* (Figs 1G, H, 6B–D). Based on other records, the morphology of the outer tepals (also call lateral tepals, Smith 1927) and the appendage of *T. labiata* are more similar to the tepals of *T. sahyadrica*. Additionally, while the inner tepals of *T. labiata* are linear to filiform (appearing as a long appendage without a tepal, Fig. 6A, B), this characteristic is different in *T. belumensis*, inner tepals obovate to spathulate with a ca. 3 cm long appendage (Figs 1H, 2F). Furthermore, the *T. belumensis* stigma morphology is nearly similar to *T. labiata* but completely different from *T. sahyadrica*. Finally, all three species have vermiform roots, with both *T. belumensis* and *T. labiata* possessing simple vermiform roots with and single axis. By contrast, *T. sahyadrica* possesses vermiform roots with clustering at the base of many stems. In summary, there is no doubt that *T. belumensis* is unique, but morphologically closest to *T. labiata* – especially with its zygomorphic flower. However, *T. belumensis* has certain other morphological characteristics that indicate a different position and relatives in *Thismia*.

As discussed above, *T. belumensis* resembles *T. labiata* (Fig. 6) – a Sumatran (Indonesia) species for which own section Labiothismia was proposed (Smith 1927) – in its growth and overall appearance (i.e., bilaterally symmetrical flowers and stamens with elongated thecae). However, it must be noted that the Labiothismia section is not recognised by Kumar et al. (2017), which instead places *T. labiata* in subsection Brunonithismia. *Thismia belumensis* also belongs to subgenus Thismia and potentially belongs to subsection Brunonithismia along with *T. labiata* (Kumar et al. 2017). However, the accuracy of this placement is uncertain, and *T. belumensis* probably cannot be included in subgeneric classification proposed by Kumar et al. (2017). Further investigation is greatly needed.

*Thismia belumensis* could also potentially belong in section Glaziocharis (Shepeleva et al. 2020). The most prominent trait of species in this section is an absence of the lateral appendage, but other traits also suggest this placement; for example, the presence of a false mitre and the absence of transverse bars inside the hypanthium, both observed in *T. belumensis*. The absence of interstaminal glands and a lateral appendage (also called a wing-like appendage) suggest that all three species’ (*T. belumensis*, *T. labiata* and *T. sahyadrica*) are closely related to species forming clade 1 in Shepeleva et al. (2020). Whether they are encapsulated within this section or form a distinct sister group must be resolved by phylogeny studies.

In conclusion, the significant differences and unique traits of *T. belumensis* – i.e., the floral tube detail, the annulus structure, the morphology of the tepals and appendages, and the stamens – compared to other *Thismia* species worldwide, strongly supports the recognition of this remarkable new species. Further study of this new species, such as, its life cycle, pollination system, and including molecular analysis, is crucial to gain new knowledge and understanding of the phylogenetic and biogeographic of *Thismia* of the world.

## Supplementary Material

XML Treatment for Thismia belumensis
